# Occlusion development after premature loss of deciduous anterior teeth: preliminary results of a 24-month prospective cohort study

**DOI:** 10.1590/2177-6709.29.1.e2423285.oar

**Published:** 2024-03-04

**Authors:** Patricia NADELMAN, Eduardo Otero Amaral VARGAS, Guido Artemio MARAÑÓN-VÁSQUEZ, Ana Lúcia VOLLÚ, Matheus Melo PITHON, Amanda Cunha Regal de CASTRO, Lucianne Cople MAIA

**Affiliations:** 1 Universidade Federal do Rio de Janeiro, Faculdade de Odontologia, Departamento de Odontopediatria e Ortodontia (Rio de Janeiro/RJ, Brazil).; 2 Universidade Estadual do Sudoeste da Bahia, Departamento de Saúde (Jequié/BA, Brazil).

**Keywords:** Tooth loss, Tooth, deciduous, Incisor, Dental occlusion, Models, dental, Digital technology, Perda de dente, Dente decíduo, Incisivo, Oclusão dentária, Modelos dentários, Tecnologia digital

## Abstract

**Objective:**

This study aimed to evaluate occlusion development after premature loss or extraction of deciduous anterior teeth, by means of a prospective cohort study.

**Methods:**

Fifteen infants and children aged 1 to 5 years old were longitudinally assessed (with loss or extraction of deciduous anterior teeth [n = 9], and without tooth losses [n = 6]). Photographs and dental casts at the baseline and after 24 months of follow-up were performed. Dental casts were scanned, and linear measurements were made on the digitalized models (missing tooth space, arch perimeter, arch length, arch width, intercanine length and intercanine width). The *t*-test was used for groups comparisons (α = 0.05).

**Results:**

Individuals’ mean age at baseline was 2.93 (± 1.18) years. No statistically significant differences were observed in the missing tooth space in the group with tooth loss during the 24 months of follow-up (*p* > 0.05). Arch perimeter, arch length, arch width, intercanine length and intercanine width did not show differences between the groups (*p* > 0.05). Qualitative photographic evaluation revealed other changes in the dental arches and occlusion, such as exfoliation and eruption of deciduous teeth, eruption of permanent teeth, self-correction or establishment of malocclusion, among others.

**Conclusion:**

The results suggest that the premature loss of deciduous anterior teeth does not affect the perimeter, length and width of the dental arches; however, other alterations that lead to malocclusion could be established.

## RESUMO

**Objetivo:**

Este estudo teve como objetivo avaliar o desenvolvimento da oclusão após perda prematura ou extração de dentes decíduos anteriores, por meio de um estudo de coorte prospectivo.

**Métodos:**

Quinze bebês e crianças de 1 a 5 anos foram avaliados longitudinalmente (com perda ou extração de dentes anteriores decíduos [n = 9] e sem perdas dentárias [n = 6]). Foram realizadas fotografias e modelos dentais no início e após 24 meses de acompanhamento. Os modelos dentários foram escaneados e medidas lineares foram feitas nos modelos digitalizados (espaço dentário perdido, perímetro da arcada, comprimento da arcada, largura da arcada, comprimento intercaninos e largura intercaninos). O teste *t* foi utilizado para comparações entre grupos (α = 0,05).

**Resultados:**

A média de idade dos indivíduos no início do estudo foi de 2,93 (± 1,18) anos. Não foram observadas diferenças estatisticamente significativas no espaço dentário perdido no grupo com perda dentária durante os 24 meses de acompanhamento (*p* > 0,05). O perímetro da arcada, comprimento da arcada, largura da arcada, comprimento intercaninos e largura intercaninos não apresentaram diferenças entre os grupos (*p*> 0,05). A avaliação fotográfica qualitativa revelou alterações nas arcadas dentárias e na oclusão, como: esfoliação e erupção de dentes decíduos, erupção de dentes permanentes, autocorreção ou estabelecimento de má oclusão, entre outras.

**Conclusão:**

Os resultados sugerem que a perda prematura de dentes anteriores decíduos não afeta o perímetro, comprimento e largura das arcadas dentárias; entretanto, outras alterações que levam à má oclusão poderiam ser estabelecidas.

## INTRODUCTION

The development of dental occlusion is a continuous process of long-term occurrence from the sixth week of intrauterine life to approximately 20 years of age.^1^ ^,^ ^2^ Knowledge about normal characteristics of dentition at different stages is essential to recognize and diagnose deviations from normality in an early stage, and to perform the appropriate treatment, when it is necessary.^3^

Deciduous teeth are the first to erupt in the oral cavity. Deciduous dentition evolution is extremely relevant, as it provides dental arches’ development from alveolar bone matrix, as well as guidance for the eruption of permanent teeth.^4^ In general, the eruption period of deciduous teeth extends from 6 months to 2.5 years of age; and, by the age of 3 years, the deciduous dentition is completely established.^4^ ^,^ ^5^

Genetic and environmental factors can influence the normal development of dental arches and occlusion.^6^ Among the environmental factors, the premature loss of deciduous teeth can be highlighted. Tooth loss is considered premature when the deciduous tooth is lost before the permanent successor has begun its active eruption, compatible with stage 6 of Nolla, in which crown formation is completed and there is less than 2/3 of root formation, evidenced through radiographic exam.^7^

Premature loss of deciduous anterior teeth can cause morphological, functional and psychosocial damage to the infant/child.^8^ It is believed that morphological consequences might include interferences in eruption of permanent successors teeth and impairment of arch integrity.^9^ However, there are gaps in the scientific literature about the real effect of premature loss of deciduous anterior teeth in dimensional arch changes, and how it affects permanent tooth alignment and occlusion establishment.^8^

The few studies found in the literature are outdated and present substantial methodological flaws, such as: lack of comparison group,^10^ absence of numerical data,^11^ non-specification of type of teeth lost,^12^ premature loss or extraction due to crowding and ectopic eruption of permanent lateral incisors,^13^ and assessment of both anterior and posterior tooth loss.^14^ ^-^ ^17^ Consequently, there is little robust scientific evidence for the clinical practice decisions. Therefore, the present cohort study aimed to evaluate changes in dental arches and occlusion development of infants and children who have suffered premature loss or extraction of deciduous incisor(s) and/or canine(s), compared to infants and children without tooth losses, during a 24-month follow-up period.

## MATERIAL AND METHODS

The report of the preliminary data of the present prospective cohort study was conducted following the STROBE (Strengthening the Reporting of Observational Studies in Epidemiology) statement.^18^

### STUDY DESIGN

The present study was an observational longitudinal prospective cohort study that aimed to evaluate dental arches and occlusion development of infants and children aged 0 to 6 years who had premature lost or extraction of deciduous incisor(s) and/or canine(s), compared to infants and children without any premature loss or extraction.

### SETTING

Research Ethics Committee from *Universidade Federal do Rio de Janeiro* (UFRJ, Brazil) approved the study protocol (approval number: 02502818.7.0000.5257, report number: 5.621.927) and an informed consent form was provided to all of guardians, allowing participation of infant/child in the study. Eligible participants who sought care at the *Centro de Vigilância e Monitoramento de Traumatismos Dentoalveolares* (CVMT) and at the Infant Clinic of the Department of Pediatric Dentistry and Orthodontics of School of Dentistry from UFRJ composed the study sample.

Participants’ recruitment period occurred between April 2019 and March 2020. The study aimed to evaluate participants at baseline (after premature loss, if any) and review them at follow-up visits 6 and, originally, 12 months later. However, due to the COVID-19 pandemic, participants were only evaluated at baseline (T0) and after at least 24 months (T1). Thus, data collection had to be carried out in two periods: April 2019 to March 2020; and November 2021 to April 2022.

### PARTICIPANTS

The inclusion criteria for the exposed group were infants and children aged from 0 to 6 years with premature loss or extraction of one or more maxillary or mandibular incisors and/or canines due to trauma, caries or neonatal teeth; good general health; complete or incomplete deciduous dentition or mixed dentition; absence of cavitated carious lesions and/or restorations; absence of non-nutritive habits, skeletal malocclusions and/or oral appliances. The same inclusion criteria were applied to non-exposed group, except that this group consisted of individuals with no tooth loss or extraction.

The exclusion criteria for both groups were infants and children with special needs, loss or extraction of deciduous posterior teeth; previous or ongoing orthodontic or orthopedic treatment; or cleft lip and/or palate. 

### VARIABLES

A trained and calibrated (ICC ranging from 0.93 to 1.00) operator (PN) performed data collection. After concluding anamnesis, participants underwent an intraoral examination, recording characteristics of the occlusion, such as: type of dentition (deciduous complete or incomplete or mixed dentition), Baume’s deciduous dental arch type (type I or II) and canines relationship (Class I, II or III). For participants with premature loss of deciduous canines or those who had no fully erupted canines, canines relationship was not classified.

Tooth loss or extraction was considered premature when it happened before physiological exfoliation time, while the permanent successor tooth has not begun its active eruption, compatible with stage 6 of Nolla, in which crown formation is completed and there is less than 2/3 of root formation, evidenced through radiographic exam.^7^

Patients from exposed group underwent radiographic examination for trauma monitoring in CVMT and consequent inclusion in the present study. The operator measured the missing tooth space (MTS), in exposed group, directly on patient’s oral cavity with a digital caliper (Absolute Digimatic Caliper, Mitutoyo, Kawasaki, Japan). MTS was determined as the linear distance between the most prominent points of interproximal surfaces of teeth adjacent to edentulous space.

Intraoral recording was carried out using intraoral photographs and study models. Frontal intraoral photographs were performed with a Canon EOS Rebel T3i digital camera, macro lens (Tokina 100/2.8 AT-X PRO D, Canon), circular flash (Macro Circular Meike Mk-14ext, Canon) and with the aid of frontal infant lip retractors. When photograph of patient’s occlusion was not possible due to lack of cooperation, intraoral photographs of dental arches out of occlusion were taken.

Upper and lower arches impressions were taken with alginate Orthoprint Tipo I (Zhermack, Badia Polesine, Italy) in standard perforated plastic (Maquira, Maringá/PR, Brazil and Morelli, Sorocaba/SP, Brazil) or aluminum trays (Tecnodent, Indaiatuba/SP, Brazil), depending on the patient’s dental arch size.

Study models were created with special orthodontic plaster (Asfer Indústria Química Ltda, São Caetano do Sul, São Paulo, Brazil) in the proportion recommended by the manufacturer. Models were prepared and cut according to proposals from the literature.^19^

Finished models were numbered in a blind way and individually scanned using an optical 3D scanner (Open Technologies, Rezzato, Lombardy, Italy). Models scanning sequence consisted of scanning the upper model, then the lower model and, lastly, the occluded models, to obtain inter-arches relation, as well as sagittal, vertical and cross-section adjustment of intercuspation.

The entire process described above - occlusion examination, MTS measurement, intraoral photographs, upper and lower arches impressions, study models preparation and models scanning - was performed at two different time points: T0 and T1.

### DATA SOURCES/MEASUREMENT

Six dental linear measurements concerning to dental arch development were considered:^20^ (1) MTS (only in the exposed group), (2) arch perimeter, (3) arch width, (4) arch length, (5) intercanine width and (6) intercanine length. For participants with premature loss of deciduous canines or those who had no fully erupted canines, intercanine width and intercanine length were not measured.

A trained and calibrated operator (PN) digitally performed dental linear measurements in a blinded way, using the Autodesk Meshmixer software (v. 3.5.474, California, United States).^21^ All digital measurements were repeated after a 1-month interval, for evaluation of method error.

### BIAS

After data collection had already started, COVID-19 pandemic broke out and interrupted the recruitment of eligible patients, since clinical activities at Department of Pediatric Dentistry and Orthodontics of School of Dentistry were suspended from March 2020 to November 2021. Thus, a non-probabilistic sample was adopted and the follow-up period for participants already included has increased from 12 to 24 months. Recruitment proposal for the subjects planned for sample pairing by gender and age. This pairing could not be guaranteed also because of COVID-19.

### STATISTICAL ANALYSIS

Statistical analysis was performed using the Jamovi software v. 2.2 (Sydney, Australia), adopting a significance level of 5%. Descriptive statistics were used to present data (absolute and relative frequencies and mean and standard deviation for dental arch measurements). A comprehensive descriptive analysis was performed through clinical evaluation and photographs of each patient comparing T0 and T1. Physiological and pathological changes in T0 and T1 were observed and highlighted in the descriptive analysis.

The Wilcoxon test was used to evaluate if there were clinical changes in MTS. The ICC was used to calculate the intra-rater repeatability. Bland-Altman test was applied to evaluate method agreement and systematic error, by estimating the proportion bias. Shapiro-Wilk test was used to verify data normality. Levene’s test was used to evaluate normality of variances. Once normality was verified, the t-test was employed to compare the changes in dental linear measurements between the exposed and non-exposed group.

## RESULTS

The intra-rater reproducibility was good. This was evidenced by ICC values ranging from 0.73 to 1.00. Bland-Altman test showed that method agreement was adequate and there was no evidence of proportion bias, which means that no systematic error was detected in measurements (Table 1).

**Table 1:** Method’s error assessments.

Measurement	ICC (95% CI)	Bland-Altman - Proportion bias Estimate (95% CI)
Space loss	0.99 (0.97 - 1.00)	0.01 (-0.18 - 0.20)
Upper arch perimeter	1.00 (0.99 - 1.00)	-0.33 (-0.71 - 0.04)
Upper arch width	0.99 (0.97 - 1.00)	-0.10 (-0.36 - 0.16)
Upper arch length	0.99 (0.98 - 1.00)	0.20 (-0.10 - 0.50)
Upper intercanine width	0.99 (0.96 - 1.00)	0.12 (-0.04 - 0.28)
Upper intercanine length	0.97 (0.90 - 0.99)	0.12 (-0.10 - 0.33)
Lower arch perimeter	1.00 (1.00 - 1.00)	-0.20 (-0.40 - 0.01)
Lower arch width	0.98 (0.94 - 0.99)	-0.22 (-0.57 - 0.12)
Lower arch length	0.83 (0.56 - 0.94)	-0.23 (-1.59 - 1.12)
Lower intercanine width	0.98 (0.93 - 0.99)	0.09 (-0.07 - 0.26)
Lower intercanine length	0.73 (0.34 - 0.90)	-0.18 (-0.63 - 0.27)

Twenty-one infants and children initiated the present cohort study; however, after interruption of clinical activities due to COVID-19 pandemic, 6 patients (28,57%) dropped out. Thus, the sample consisted of 15 infants and children distributed into exposed group(n = 9) and non-exposed group (n = 6), with mean age of 2.93 (± 1.18) years at baseline. Regarding the sex, 6 patients (40%) were boys, and 9 patients (60%) were girls (Table 2). All premature losses occurred as a result of dentoalveolar trauma.

**Table 2:** Sample characteristics.

Characteristics	Exposed (n = 9)	Non exposed (n = 6)	Total sample (n = 15)
	n (%)	n (%)	n (%)
Group age (years)			
1	1 (11.1)	1 (16.7)	2 (13.3)
2	2 (22.2)	2 (33.3)	4 (26.7)
3	1 (11.1)	2 (33.3)	3 (20.0)
4	4 (44.4)	1 (16.7)	5 (33.3)
5	1 (11.1)	0 (0.0)	1 (6.7)
Sex			
Male	5 (55.6)	1 (16.7)	6 (40.0)
Female	4 (44.4)	5 (83.3)	9 (60.0)
Dentition			
Incomplete deciduous	2 (22.2)	3 (50.0)	5 (33.3)
Complete deciduous	6 (66.7)	3 (50.0)	9 (60.0)
Mixed	1 (11.1)	0 (0.0)	1 (6.7)
Baume’s deciduous dental arch type			
Type I	8 (88.9)	6 (100.0)	14 (93.3)
Type II	1 (11.1)	0 (0.0)	1 (6.7)
Canines relationship			
Class I	7 (77.7)	3 (50.0)	10 (66.7)
Class II	0 (0.0)	0 (0.0)	0 (0.0)
Class III	0 (0.0)	0 (0.0)	0 (0.0)
Canines didn’t fully erupt	1 (11.1)	3 (50.0)	4 (26.6)
Loss of canine	1 (11.1)	0 (0.0)	1 (6.7)
MTS measurement, mean dif (mm)	0.2	-	-

MTS = missing tooth space.

Concerning occlusion characteristics at the beginning of the study, 5 participants (33.3%) presented incomplete deciduous dentition, 9 participants (60%) presented complete deciduous dentition and just 1 patient (6.7%) presented mixed dentition with one permanent first molar erupted (Table 2). Almost the entire sample was composed of patients with Baume’s deciduous dental arch type I (93.3%), being 8 patients from exposed group and 6 from non-exposed group. Only 1 patient from exposed group presented the type II arch. Regarding canines relationship, 66.7% of the sample presented Class I, while the other 26.6% didn’t have deciduous canines fully erupted, and 6.7% had premature loss of canine. There was no statistical difference in the MTS of exposed group comparing T0 with T1 (*p* = 0.938). The space remained unchanged, without loss or gain of space.

Considering dental arches, there was no statistical difference between the groups at the beginning of the study (*p* > 0.05) (Table 3). Regarding spatial changes in dental arches after the 24-month follow-up, results showed no statistical differences in the upper and lower arches between exposed and non-exposed groups (*p* > 0.05) (Table 4). 

**Table 3:** Comparisons between the exposed and non-exposed group at T0.

Measurements (mm)	Exposed (n = 9) mean ± SD	Non exposed (n = 6) mean ± SD	P-value
Space loss	8.7 ± 2.2	---	---
Upper arch perimeter	73.3 ± 9.7	67.1 ± 11.6	0.282
Upper arch width	38.8 ± 3.5	36.9 ± 3.2	0.307
Upper arch length	22.6 ± 4.3	19.4 ± 4.5	0.190
Upper intercanine width	30.2 ± 1.9	29.7 ± 2.0	0.646
Upper intercanine length	9.0 ± 2.0	7.9 ± 0.9	0.233
Lower arch perimeter	65.4 ± 8.8	59.4 ± 12.5	0.293
Lower arch width	34.3 ± 2.7	32.2 ± 4.0	0.231
Lower arch length	19.2 ± 2.6	16.3 ± 4.9	0.226
Lower intercanine width	23.6 ± 1.4	22.5 ± 1.3	0.192
Lower intercanine length	4.9 ± 0.8	4.6 ± 1.2	0.546

SD = standard deviation.

**Table 4:** Comparison of changes (T1-T0) in dental linear measurements of dental arches between exposed and non-exposed groups.

Measurements (mm)	Exposed (n = 9) mean dif ± SD	Non exposed (n = 6) mean dif ± SD	P-value
Space loss	1.1 ± 2.2	---	---
Upper arch perimeter	1.5 ± 1.6	0.1 ± 2.1	0.156
Upper arch width	1.6 ± 1.0	1.4 ± 1.0	0.672
Upper arch length	-0.4 ± 2.2	-0.6 ± 1.0	0.886
Upper intercanine width	1.5 ± 1.2	0.7 ± 1.6	0.342
Upper intercanine length	-0.1 ± 1.3	0.0 ± 1.0	0.857
Lower arch perimeter	0.8 ± 1.9	0.7 ± 2.5	0.915
Lower arch width	1.2 ± 0.8	1.3 ± 1.2	0.860
Lower arch length	1.1 ± 3.4	0.7 ± 0.9	0.790
Lower intercanine width	1.0 ± 1.0	0.5 ± 1.2	0.429
Lower intercanine length	1.1 ± 0.7	0.8 ± 0.4	0.334

SD = standard deviation.

**Figure 1:**
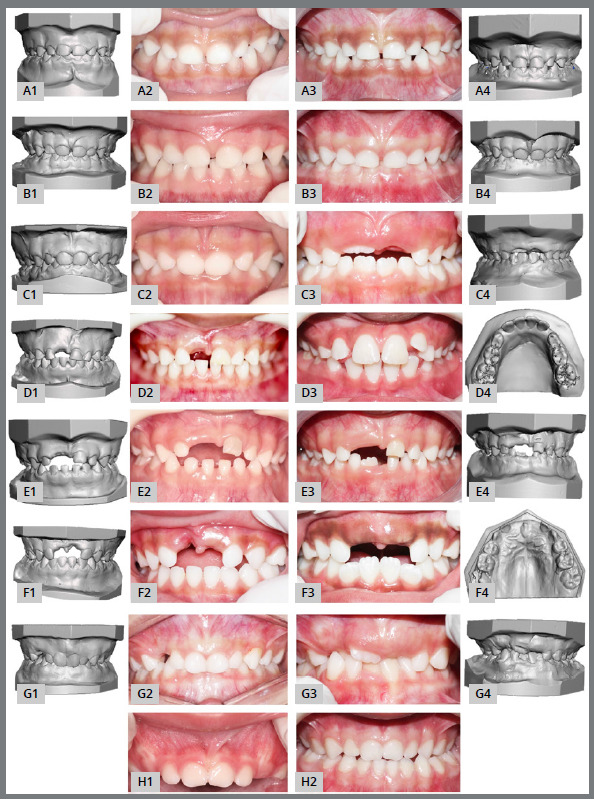
Photographs and digital models of patients from exposed and unexposed groups at T0 and T1, to illustrate changes occurred after 24 months. A1, B1, C1, D1, E1, F1, G1 = Digital models from T0; A2, B2, C2, D2, E2, F2, G2, H1 = Intraoral photographs from T0; A3 and A4 = intraoral photograph and digital models from T1 demonstrating overbite decrease by the eruption of deciduous upper second molars (#55 and #65); B3 and B4 = intraoral photograph and digital models from T1 showing overbite increase; C3 and C4 = intraoral photograph and digital models from T1 showing deciduous teeth (#51, #61, #71 and #81) exfoliated and permanent teeth (#11, #31 and #41) eruption; D3 and D4 = intraoral photograph and digital models from T1 presenting premature loss of lower deciduous canines (#73 and #83) due to ectopic eruption of permanent lateral incisors (#32 and #42); E3 and E4 = intraoral photograph and digital models from T1 presenting self-correction of anterior open bite; F3 and F4 = intraoral photograph and digital models from T1 showing primary crowding in lower permanent incisors (#31, #32, #41 and #42), opening of MTS by the initiation of permanent successors eruption (#11 and #21), and ectopic eruption of permanent first molar (#26); G3 and G4 = intraoral photograph and digital models from T1 presenting especially primary crowding, establishment of anterior crossbite, and lack of space for eruption of permanent teeth; H2 = establishment of posterior crossbite and deciduous teeth eruption.

Although there were no statistically significant differences in dental arches and occlusion development of infants and children with or without premature loss of deciduous anterior teeth, it was possible to observe physiological and pathological changes in dental arches and occlusion after 24 months follow-up through photographic recording and study models. Through a qualitative analysis, it could be noticed that both the exposed group and the non-exposed group showed changes, such as: deciduous teeth eruption and exfoliation; permanent teeth eruption; emergence of primary crowding; ectopic eruption of permanent first molars; self-correction of anterior open bite; establishment of anterior open bite, anterior and posterior crossbite; premature loss of deciduous canines due to ectopic eruption of permanent lateral incisors; overbite increase; overbite decrease by the eruption of deciduous second molars; opening of MTS by the permanent successor eruption (Fig 1).

## DISCUSSION

Supervising the eruption and development of deciduous and mixed dentition represents an essential component for the evolution of a permanent dentition with stable, functional and esthetic occlusion.^1^ This supervision provides early detection of genetic and environmental factors that may result in potential occlusion problems and future treatment needs.^6^ Premature loss of deciduous teeth is a critical environmental factor identified during occlusion monitoring that can cause functional and morphological problems for dentition.^8^

It is well known that there is an amount of space decrease after premature loss of deciduous posterior teeth.^22^ ^-^ ^24^ However, there is a gap in the scientific literature about consequences of premature loss of deciduous anterior teeth on dental arches integrity.^25^ Hence, the present study aimed to evaluate dental arches and occlusion development of infants and children who have suffered premature loss of deciduous incisor(s) and/or canine(s), compared to infants and children without premature losses, through a 24-month follow-up period.

Despite the lack of scientific evidence, there seems to be a consensus in dentistry, based on clinical experiences, that premature loss of deciduous anterior teeth would not require treatment, as it does not cause space loss.^9^ In an attempt to support clinical decisions on scientific evidence, the present study provides preliminary results indicating that there are no space changes in dental arches after this premature loss. Taking into account the limitations of a small sample size, and the number of dropouts due to the COVID-19 pandemic, current results agrees with the only classic clinical study published,^14^ which found absence of space closure after premature loss of deciduous anterior teeth. 

Considering possible causes for no space changes, it can be highlighted that between 3 and 6 years of age, deciduous dentition is generally stable, with no significant variations in individual tooth positioning or in the transverse and sagittal interarch relationships.^26^ Since transversal dimensions of deciduous dental arches barely change from 3 to 6 years of age,^27^ this may have contributed to maintenance of arch dimensions.

On the other hand, transition from deciduous to permanent dentition extends over a prolonged period of child development - from about 6 years of age, with the mixed dentition, characterized by replacement of the dentition, facial growth, and dimensional changes of the dental arches.^28^ In this context, it is important to remember that at the study final evaluation, almost half of the sample was with complete deciduous dentition, and the other half was in the first transitional period of mixed dentition, characterized by the eruption of first permanent molars and/or exfoliation of deciduous incisors followed by eruption of permanent incisors. In addition, the potential for increased intercanine width and/or space loss during eruption of the permanent incisor, as a compensatory mechanism, must be considered.

Even though there were no statistically significant differences in dental arches of infants and children either with or without premature loss of deciduous anterior teeth, regardless of the type of dentition, several changes could be observed in dental arches and occlusion of both groups during the monitoring period. Regarding the changes that could not be confirmed by statistical analysis, the following can be emphasized: deciduous teeth eruption and exfoliation; permanent teeth eruption; emergence of primary crowding; ectopic eruption of permanent first molars; self-correction of anterior open bite; establishment of anterior open bite, anterior and posterior crossbite; premature loss of deciduous canines due to ectopic eruption of permanent lateral incisors; overbite increase; overbite decrease by the eruption of deciduous second molars; opening of MTS by the permanent successor eruption. 

Furthermore, some dental arches didn’t present notable clinical changes from the beginning to the end of the study, corroborating the theory^26^ ^,^ ^27^ that between complete deciduous dentition and the beginning of the mixed dentition, dental arches transverse dimensions remain stable, without significant clinical changes. It can be observed that occlusion development occurred in both groups in a similar way. Changes or maintenance of dental arch dimensions and occlusion progression happen regardless of exposure to premature loss of deciduous anterior teeth, and this corroborates the result presented in the statistics.

In addition, certain factors could increase the possibility of space loss, including Baume’s deciduous dental arch type II, presence of non-nutritive habits, deciduous canines erupted at the loss moment, and amount of teeth lost.^8^ A greater possibility of space loss might be observed if there is no spacing (primate and generalized spaces) between the deciduous teeth or evidence of an arch-length inadequacy in the anterior region before tooth loss.^1^ As most of the sample had Baume arch type I, and deciduous canines already erupted at the time of loss, the potential of space loss was smaller. In addition, presence of non-nutritive habits was an exclusion criterion, and then it didn’t influence dental arches’ development. This fact also significantly reduced the sample size, as the majority of infants had non-nutritive habits such as pacifier, thumb, or bottle sucking.

Regarding the number of teeth lost or extracted, White^29^ reported that consequences related to arch space loss are determined by the amount of incisors and canines lost. The author proposed that in the event of losing only one central incisor at a young age, significant alterations in the dental arch are unlikely to occur, except for a potential minor misalignment in the midline. If both central incisors are lost, it has been documented that there is no notable impact on the overall arch perimeter, but there is a chance that deleterious habits like tongue thrusting may develop. Once central and lateral incisors are prematurely lost, the potential consequences of acquiring deleterious oral habits may be more frequent, along with other outcomes such as extrusion of lower incisors to compensate for the lack of contact with opposing teeth.

Since space loss is usually minimal, unless the tooth or teeth are lost at a very young age, space maintenance after premature loss of deciduous anterior teeth is generally not necessary. Besides that, if the tooth is lost at a very young age, the infant/child has neither deciduous second molars for anchorage of the space maintainer, nor sufficient maturity to receive an appliance.^5^ Therefore, literature suggests that space maintainer treatments should start at the age of 5.^5^

As noted, the main limitation of this study was the small sample size. It is worth mentioning that besides the suspension of subject recruitment and data collection for almost two years due to the COVID-19 pandemic, clinical measurement and arches’ impression in infants are a challenge, since all of them present definitively negative behavior,^30^ without cooperation to perform the clinical steps.

Concerning measurement instruments chosen for the study, digital caliper was selected for clinical evaluation of missing tooth/teeth space due to its high precision, fast reading and facility for statistical control.^31^ The only difficulty experienced during the clinical measurement was that infants/children showed fear of the sharp point of the digital caliper. This shows that pediatric dentist and orthodontist need to use behavior management techniques to measure space. In turn, study model measurements were performed in digital software since technology has become part of dental practice, in which dental plaster models have been replaced by digital models to facilitate diagnosis and treatment planning.^32^ ^,^ ^33^ Besides that, digital measurements of deciduous dentition have apparently high accuracy level, comparable to direct measurement of plaster models.^34^

## CONCLUSION

The present study concluded that there were no statistical differences over 24 months in dental arches and occlusion development of infants and children with premature loss of deciduous anterior teeth, compared to infants and children without losses. However, physiological and pathological changes could be observed through clinical examination in both groups, which were not evidenced by statistical analysis. Therefore, periodic and long-term clinical follow-up of infants and children in dentition and occlusion development is recommended.
